# Combined effects of border irrigation and super-absorbent polymers on enzyme activity and microbial diversity of poplar rhizosphere soil

**DOI:** 10.1371/journal.pone.0303096

**Published:** 2024-05-07

**Authors:** Fangchun Liu, Ruonan Zhuang, Yanhui Qiao, Dawei Jing, Yufeng Dong

**Affiliations:** 1 Institute of Resource and Environment, Shandong Academy of Forestry, Jinan, China; 2 College of Ecology, Resources and Environment, Dezhou University, Dezhou, China; Institute for Biological Research, University of Belgrade, SERBIA

## Abstract

Fast-growing poplar plantations are considered a great benefit to timber production, but water availability is a key factor limiting their growth and development, especially in arid and semi-arid ecosystems. Super-absorbent polymers facilitate more water retention in soil after rain or irrigation, and they are able to release water gradually during plant growth. This study aimed to examine the effects of reduced irrigation (60% and 30% of conventional border irrigation) co-applied with super-absorbent polymers (0, 40 kg/ha) on root exudates, enzyme activities, microbial functional diversity in rhizosphere soil, and volume increments in poplar (*Populus euramericana* cv. ‘Neva’). The results showed that 60% border irrigation co-applied with super-absorbent polymers significantly increased the content of organic acids, amino acids and total sugars in the root exudates, and the activities of invertase, urease, dehydrogenase, and catalase in the rhizosphere soil in comparison to conventional border irrigation without super-absorbent polymers. Meanwhile, this treatment also enhanced the average well-color development, Shannon index, and McIntosh index, but decreased the Simpson index. Additionally, the average volume growth rate and relative water content of leaves reached their maximum using 60% irrigation with super-absorbent polymers, which was significantly higher than other treatments. However, using 30% irrigation with super-absorbent polymers, had a smaller effect on rhizosphere soil and volume growth than 60% irrigation with super-absorbent polymers. Therefore, using an appropriate water-saving irrigation measure (60% conventional border irrigation with super-absorbent polymers) can help to improve enzyme activities and microbial diversity in the rhizosphere soil while promoting the growth of poplar trees.

## Introduction

Poplar, which is currently the major afforestation tree species of rapid-growing and high-yield forests in China, plays an important role in wood-production, urban greening, bioenergy, and desertification control [1, 2]. Currently, flood irrigation is the main strategy in the water management of poplar plantations. Flood irrigation not only causes various environmental problems, such as soil compaction and poor permeability, but it also leads to a substantial water waste, soil erosion, and nutritional losses, thus posing a potential threat to underground water via its contamination. Moreover, water resource shortages are a serious problem in the northern regions of China. The north contains 64% of the land area, but possesses only 20% of the country’s water resources [3]. This situation is gradually becoming a major problem and it is limiting agricultural and forestry development [4, 5]. Furthermore, the problem will be amplified in the coming decades because of climate change, by which may lead to reduced rainfall, rising temperatures, and increasing evapotranspiration [6, 7]. Therefore, it is essential to improve soil moisture conservation, develop water-saving technologies, and increase water use efficiency to sustainably develop the Chinese agroforestry sector in the future. To do this, the traditional flood irrigation system urgently needs to be replaced by a water-saving strategy in poplar plantations via appropriate irrigation measures or new water retention materials.

Border irrigation is an irrigation measure that is low- cost, simple to operate and easily popularized [8]. Our previous studies have indicated that border irrigation maintains yield but requires only 20% of the water provided by traditional flood irrigation, which is prone to poor soil permeability, soil water-fertilizer-air-heat disharmony, and serious water waste [9]. Lv et al. [10] found that border irrigation with a border width of 2.8 m and a border length of 60 m resulted in higher water use efficiency as well as lower nitrate-nitrogen content in the deep soil. Other research showed [11] that excessive border length and width leads to lower irrigation water use efficiency and may affect the quality of irrigation water in border irrigation. Applying soil additives to improve water retention is one proven, simple and effective approach for further water savings [12]. During the last several decades, super-absorbent polymers have been extensively studied and shown to have special attributes due to their three-dimensional structure. When super-absorbent polymers are integrated into soil, they retain a large amount of water and nutrients, which are subsequently released according to the requirements of the growing plants. Consequently, super-absorbent polymers have been widely used in agroforestry water conservation and ecological restoration efforts [3, 7].

Most studies on super-absorbent polymers in agroforestry have only evaluated and compared physicochemical characteristics [12], and the effects on soil characteristics and plant growth [5]. This research has indicated that the application of super-absorbent polymers can reduce soil bulk density and soil water permeability, and help to protect soil organic matter [7]. Additionally, the combined use of irrigation and super-absorbent polymers has been reported for corn and Chinese cabbage cultivation [4, 13]. However, less information is available on the effect of border irrigation with super-absorbent polymers addition on soil biological characteristics of poplar plantations, especially in respect to enzyme activity and microbial diversity in poplar rhizosphere soil. The rhizosphere is the soil compartment profoundly modified by the release of root exudates consisting of low molecular weight organic acids, sugars, and more complex chemical molecules [14]. Root exudates enhance plant nutrient uptake, sustain a larger and more active microbial activity, and influence the composition of the rhizosphere microbial communities [14]. Soil enzymatic activities are sensitive biomarkers of any natural and anthropogenic disturbance [15, 16]. Soil enzymes are vital to catalysis of several crucial reactions that are necessary for soil microorganisms, as well as for the stabilization of organic matter formation, organic waste decomposition, and nutrient cycling [17–19]. At the same time, soil microbial community functional diversity is one of the most important microbial parameters in soil and it has been regarded as a possible indicator of soil quality [6]. Root exudates have an obvious influence on soil enzyme activities and microbial diversity [20]. Previous findings clearly showed that enzyme activities in the rhizosphere soil may well serve as indicators of microbial diversity [21]. Xu et al. [22] reported that the Shannon index and Simpson indices of the treatments applied with water retention agent were significantly higher than those of CK (no application of water retention agents) in the anthesis and harvest stages, indicating that the application of water retention agent could improve the soil microbial diversity of winter wheat in saline-alkali land. Yu et al. [23] found that the soil conditioners made of water retention agents, organic fertilizers and microbial inoculant could effectively increase the activities of soil catalase, cellulase, sucrase, urease and alkaline phosphatase, and enhance the abundance and diversity of soil bacterial communities. Therefore, soil enzyme activities and microbial diversity can represent important indexes for evaluating the sustainable development of forest land and can be frequently measured for the purpose of providing immediate and accurate information about small changes in soils [24].

In our study, we explored the effect of border irrigation co-applied with super-absorbent polymers on the root exudates, enzyme activity, and microbial community functional diversity of rhizosphere soil and on poplar growth in northern China. We hypothesized that border irrigation co-applied with super-absorbent polymers would increase enzyme activities and microbial diversity in rhizosphere soil and result in increased tree growth. The purpose was to determine the feasibility of implementing border irrigation and super-absorbent polymers for increasing the growth of poplar, with the aim of providing a better way to foster soil water-saving and high yield in poplar plantations.

## Materials and methods

### Ethics statement

This research did not involve human or other animal subjects. For soil sample collections, we collected the minimum number of specimens necessary to ensure that appropriate vouchers were obtained. The field studies did not involve endangered or protected species. Permission to work in a poplar plantation located in Jinan City was obtained through a cooperative agreement between Shandong Academy of Forestry and Jinan State-owned Nursery.

### Site description and plant material

A field experiment was carried out in a poplar plantation located in a state-owned northern suburb of the city of Jinan, Shandong province, north China (36°40′N latitude, 117°00′E longitude). The site has a warm temperate zone continental monsoon climate with four distinct seasons; average temperature and rainfall are 14°C and 650–700 mm, respectively. The main physicochemical characteristics of the 0–40 cm soil layer in the research site are shown in Table 1.

**Table 1:** Main physicochemical characteristics of the 0–40 cm-deep soil at the research site.

Soil classification	Organic matter (g/kg)	Available nitrogen (mg/kg)	Available phosphorus (mg/kg)	Available potassium (mg/kg)	pH	>0.25mm aggregate (%)	Bulk density (g/cm^3^)
Entisols	7.86	19.08	14.23	46.90	8.37	49.36	1.56

Super-absorbent polymers, a cross-linked copolymer of acrylamide and potassium acrylate, were provided by the Beijing Hanlisorb Polywater Hi-Tech. Co. Ltd. Their parameter characteristics are shown in Table 2.

**Table 2:** Some selected characteristics of super absorbent polymers.

Main materials	Types	Particle size range (mm)	Color	Water absorbency (g g^−1^)	pH^1^	Electrical conductivity (μs cm^−1^)
Cross-linked copolymer of acrylamide and K acrylate	Cationic	1.6 to 4.0	White	350	7.19	106.8

Note:

^1^Ratio of super-absorbent polymers to water is 1:1000.

Regular amounts of urea, superphosphate, and potassium sulfate were applied: 197.3 kg/ha for N, 67.7 kg/ha for P_2_O_5_, and 50.5 kg/ha for K_2_O, as first applied in 2021; these fertilizer dosages increased by 10% each year thereafter as the stand age increased. The poplar ‘I-107’ (*Populus × euramericana cv*. ‘Neva’) had been planted five years earlier using a distance of 5 m between rows and 2.5 m within rows. The experimental trees were uniform, and the average (± standard deviation, SD) tree height and stem DBH (diameter at breast height of 1.3 m) were 12.39 ± 0.46 m and 11.92 ± 0.43 cm, respectively. These poplar trees are managed carefully and grown on a short rotation (7–8 years), mainly for pulpwood production.

### Experimental design and irrigation treatment

The experiment used a randomized complete block design, with five treatments and three replications. Fifteen plots were established, and every replication for each treatment included a plot with 30 trees arrayed in five rows. The innermost 12 trees, which were identified as representative of the plot mean, were used for detailed measurements. Immediately after the leaves had fully unfolded, five treatments were applied to the poplar trees at the start of the growing season on April 10, 2021. The treatments were as follows: (1) CK (conventional border irrigation), based on the horizontal distribution of the poplar root system [25]; specifically, the border width for irrigation was set at 1.0 m and the irrigation quota was 720 m^3^/ha, with 120 m^3^/ha per month from April to September; (2) BI_60_, which amounted to 60% of the conventional border irrigation quantity; (3) BI_30_, which amounted to 30% of the conventional border irrigation quantity; (4) BI_60_+SAP,which was BI_60_ applied together with super-absorbent polymers of 40 kg/ha, and (5) BI_30_+SAP, which was BI_30_ applied together with super-absorbent polymers of 40 kg/ha.

At the first irrigation, a circular ditch that had a depth of 40 cm was prepared 60 cm away from the tree trunk. The super-absorbent polymers (50 g/tree) were mixed with the soil at 1:10 (v/v), put into the soil layer at a depth of 20–40 cm and then covered with surface soil. The irrigation time was determined based on conventional border irrigation methods. For each irrigation, the reduced irrigation treatment was controlled by its corresponding proportion of irrigation time, and the irrigation amount was measured to the nearest 0.001 m^3^ with a water meter. In addition, the super-absorbent polymers were only used once, in 2021, and the amount and method of irrigation in 2022 and 2023 were the same as those applied in 2021.

### Rhizosphere soil sampling and analysis

During late October 2023, rhizosphere soil at a distance of 60 cm from the trunk was collected following the procedures described by Wang and Zabowski [26]. Fifteen soil samples were collected from the innermost 12 trees in every plot, then mixed evenly as a composite soil sample with three replications. Root exudates in the rhizosphere soil were measured according to the method described by Klein et al. [27]. Briefly, the soil was shaken off the roots at a distance of 60 cm from the trunk and then carefully washed the roots free of fritted clay with 400 ml of distilled water four separate times. The wash solution was filtered using a 0.45 μm membrane filter and the filtered exudates were dried by rotary evaporation at 40°C, dissolved in 30 ml of distilled water and 30 μL of chloroform. The concentrated filtrates were separated into organic acid, sugar and amino acid fractions. Eight ml of the concentrate were passed successively through cation and anion exchange resins. The amino acids were eluted from a Dowex 50w × 8 cation exchange column with 2 N NH_4_OH. The carboxylic acids were eluted from a Dowex 1 × 8 anion exchange column with 5 N formic acid. The neutral solution contained the sugars. Soil enzymatic activities were determined in triplicate air-dried samples according to the method developed by Guan [15]. Briefly, invertase activity was measured using 8% sucrose as a substrate and then incubated at 37°C for 24 h to determine the produced glucose using a colorimetric method. Urease activity was measured using 10% urea solution as substrate, and the soil mixture was incubated at 37°C for 24 h; the produced NH_3_-N was determined with the colorimetric method. Dehydrogenase activity was determined using 1% triphenyltetrazolium chloride (TTC) as a substrate and then incubated in the dark at 37°C for 24 h, measuring the produced triphenylformazan (TPF) by spectrophotometry. Catalase activity was determined using 0.3% H_2_O_2_ as a substrate, shaken for 20 min and the filtrate was titrated with 0.1 M KMnO_4_.

Soil microbial communities are spatially dependent and highly responsive to environment changes [28], and the diversity of soil microbial community is closely related to the function and structure of the ecosystem [29]. Functional diversity of the soil microbial community was determined using the method of Garland and Mills [30]. The McIntosh index, Simpson index, and Shannon index were used to describe soil microbial diversity:

$$\text{McIntosh index}\;:U=\sqrt{\left(\sum_{i=1}^{n}P_{i}^{2}\right)}$$ (1)

$$\text{Shannon diversity index:}\;H=-\sum_{i}^{n}P_{i}\times \text{ln}P$$ (2)

$$\text{Simpson diversity index:}\;\lambda =\sum S_{i}^{2}$$ (3)

where *P*_i_ is every reaction well subtracting the absorbance value of the control well and then dividing by the summed color absorbance value of 31 wells; *S*_i_ is the ratio of the activity on each substrate (OD_i_) divided by the sum of activities on all substrates (ΣOD_i_) [31].

### Relative water content and volume growth rate

During mid-September 2023, the relative water content in leaves was measured according to Eneji et al. [32], using samples selected from mature leaves on the sunny side of every tree.

Individual tree height was measured by the tangent method, and tree DBH by a ruler with a 0.5-mm accuracy, at the start of the experiment (April 10, 2021) and at the end of the short rotation period (October 27, 2023). Tree volume was calculated according to Eq (4) [25]:

$$V=3.14d^{2}hf/4\;\left(f=0.42\right)$$ (4)

where *h* and *d* stand for tree height (m) and DBH (cm), respectively. Then, the average volume growth rate was calculated using Eq (5) [33]:

$$P_{v}=\frac{(V_{2}-V_{1})\times 200}{(V_{2}+V_{1})\times n}$$ (5)

where *P*_*v*_ stands for the average growth rate (%) in tree volume; *n* stands for the interval years between two measurements; *V*_l_ and *V*_2_ stand for the tree volume before and after *n* years (m^3^), respectively.

### Statistical analysis

The data were analyzed as a completely randomized design. An analysis of variance (ANOVA) evaluated the effects of the five irrigation treatments on the root exudate, enzyme activity, and microbial functional diversity in rhizosphere soil and poplar growth. When the ANOVA revealed a significant difference among the treatments, the least significant difference test was used to detect differences between the individual treatment-level means. All statistical analyses were performed at a significant level of *P* < 0.05. ANOVA and multiple comparisons were performed using SPSS software (version 23.0; SPSS Inc., Chicago, Illinois, USA).

## Results

### Root exudates

With the application of super-absorbent polymers, the organic acid content clearly increased, and reached a maximum in the BI_60_+SAP treatment, increasing by 13.40%, 42.26%, 63.10%, and 18.15% in comparison with the CK, BI_60_, BI_30_ and BI_30_+SAP treatments, respectively (Table 3). Total sugar content in the BI_60_+SAP treatment significantly increased compared to CK, BI_60_, BI_30_ and BI_30_+SAP treatments. Additionally, the amino acid content of BI_60_+SAP was also markedly higher than that of the other treatments. The results showed that the application of super-absorbent polymers significantly increased organic acids, total sugar and amino acids in the exudates of poplar rhizosphere soil, with the 60% conventional irrigation co-applied with super-absorbent polymers being the most remarkable result.

**Table 3:** Effect of different irrigation treatments on the root exudate contents in the rhizosphere soil of a poplar plantation (mean ± SD).

Treatment	Organic acid (μg kg^-1^)	Total sugar (mg kg^-1^)	Amino acid (mg kg^-1^)
CK	42.60±0.76 b	17.42±0.47 b	413.71±10.98 b
BI_60_	33.96±1.52 c	12.65±0.73 d	369.23±12.45 c
BI_30_	29.62±0.95 d	10.51±0.35 e	318.12±7.29 d
BI_60_+SAP	48.31±1.27 a	19.85±0.69 a	452.68±8.96 a
BI_30_+SAP	40.89±2.06 b	15.76±0.50 c	387.55±15.82 bc

Note: Means followed by same lowercase letter within each column were not significantly different among treatments (*P*>0.05).

### Enzyme activities in the rhizosphere soil

The activities of invertase, urease, dehydrogenase, and catalase in BI_60_ and BI_30_ treatments were markedly lower than those of CK, whereas the corresponding enzyme activities in the BI_60_+SAP and BI_30_+SAP treatments were increased when super-absorbent polymers were co-applied (Table 4). Furthermore, the activities of all four enzymes in the BI_60_+SAP treatment were evidently higher than those in other treatments, exhibiting increases of 21.52%, 7.65%, 18.07%, and 20.87% in the activities of invertase, urease, dehydrogenase and catalase compared with those of CK, respectively. Thus, reduced irrigation resulted in decreased activities of enzymes in the rhizosphere soil of the poplar plantation; however, it was the 60% irrigation coupled with super-absorbent polymers application that significantly increased soil enzyme activities.

**Table 4:** Effects of different irrigation treatments on the enzyme activities in the rhizosphere soil of a poplar plantation (mean ± SD).

Treatment	Invertase (mg glucose g^−1^ h^−1^)	Urease (mg NH_3_-N g^−1^ h^−1^)	Dehydrogenase (mg kg^−1^ 24h^−1^)	Catalase (ml 0.1 M KMnO_4_ g^−1^ h^−1^)
CK	3.02±0.15b	1.83±0.07b	61.83±1.97b	1.15±0.08c
BI_60_	2.06±0.10d	1.62±0.05c	58.70±3.02c	0.97±0.05d
BI_30_	1.73±0.08e	1.45±0.05d	46.62±1.35d	0.85±0.02e
BI_60_+SAP	3.67±0.19a	1.97±0.03a	72.98±2.16a	1.39±0.04a
BI_30_+SAP	2.58±0.21c	1.81±0.02b	59.78±2.92bc	1.30±0.05b

Note: Means followed by same lowercase letter within each column were not significantly different among treatments (*P*>0.05).

### Microbial community functional diversity

**Fig 1:**
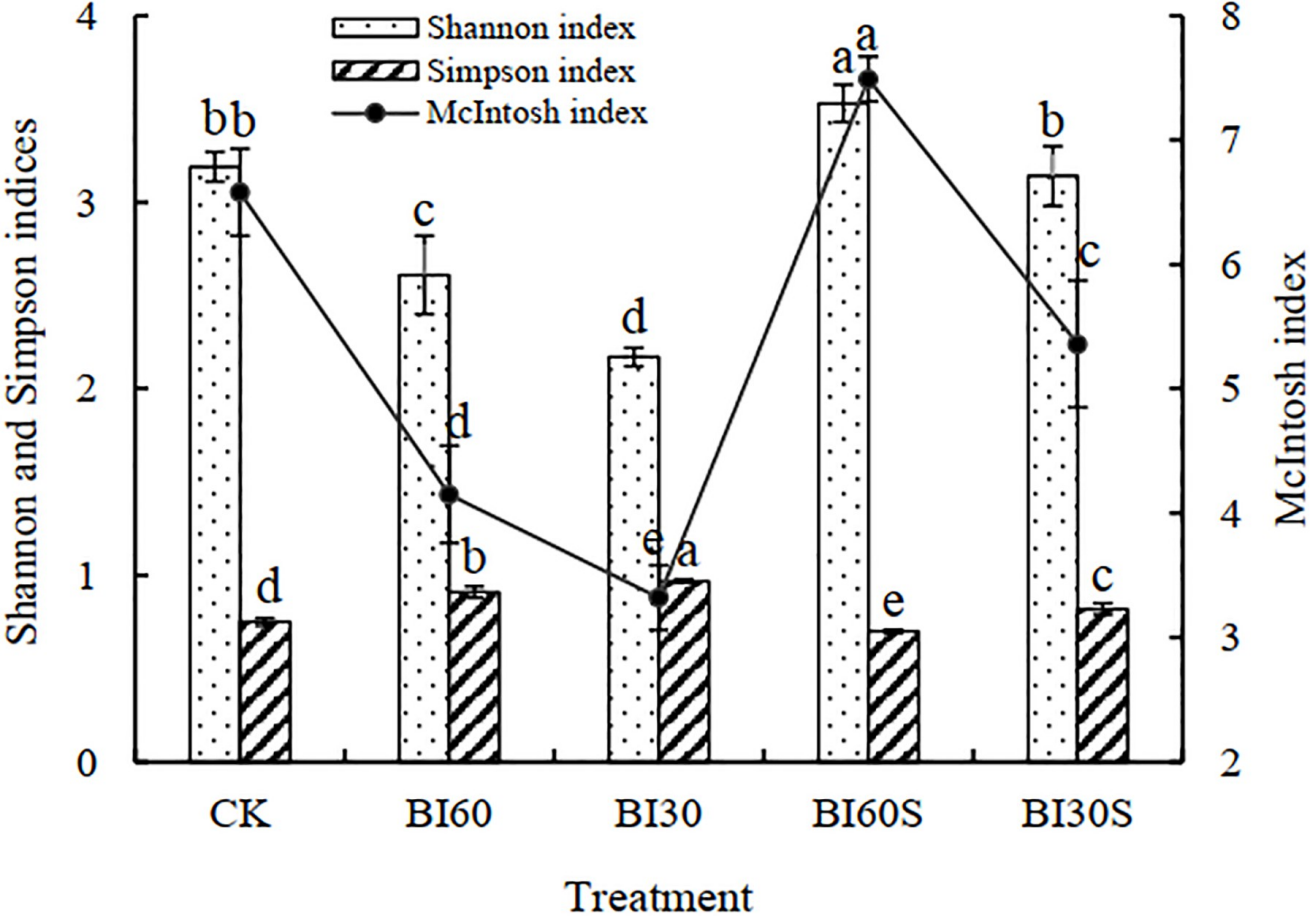
Effects of different irrigation treatments on the diversity index of microbes in the rhizosphere soil of a poplar plantation. Bars are means, and error bars are standard deviations (n = 3). Means followed by same lowercase letter within each column were not significantly different among treatments (*P*>0.05).

The microbial diversity in the rhizosphere soil of poplar under the different irrigation treatments were respectively expressed by functional diversity indices. The McIntosh index of various treatments exhibited a variation pattern of BI_60_+SAP > CK > BI_30_+SAP > BI_60_ > BI_30_, and the pairwise differences among the treatments were all significant (Fig 1). After applying the super-absorbent polymers, the Shannon index of BI_30_+SAP was similar to that of CK. The Shannon index reached its maximum in BI_60_+SAP, which significantly increased by 10.66%, 35.25%, 62.67%, and 12.42% over the treatments of CK, BI_60_, BI_30_, and BI_30_+SAP, respectively. Moreover, the Simpson index showed a different pattern to both the Shannon and McIntosh indexes, such that the Simpson index of BI_60_+SAP was evidently decreased in comparison to the other treatments. These results indicated that the 60% conventional irrigation co-applied with super-absorbent polymers greatly increased the Shannon index and McIntosh index of poplar rhizosphere soil, but it significantly reduced the Simpson index value.

### Volume growth rate and relative water content

**Fig 2:**
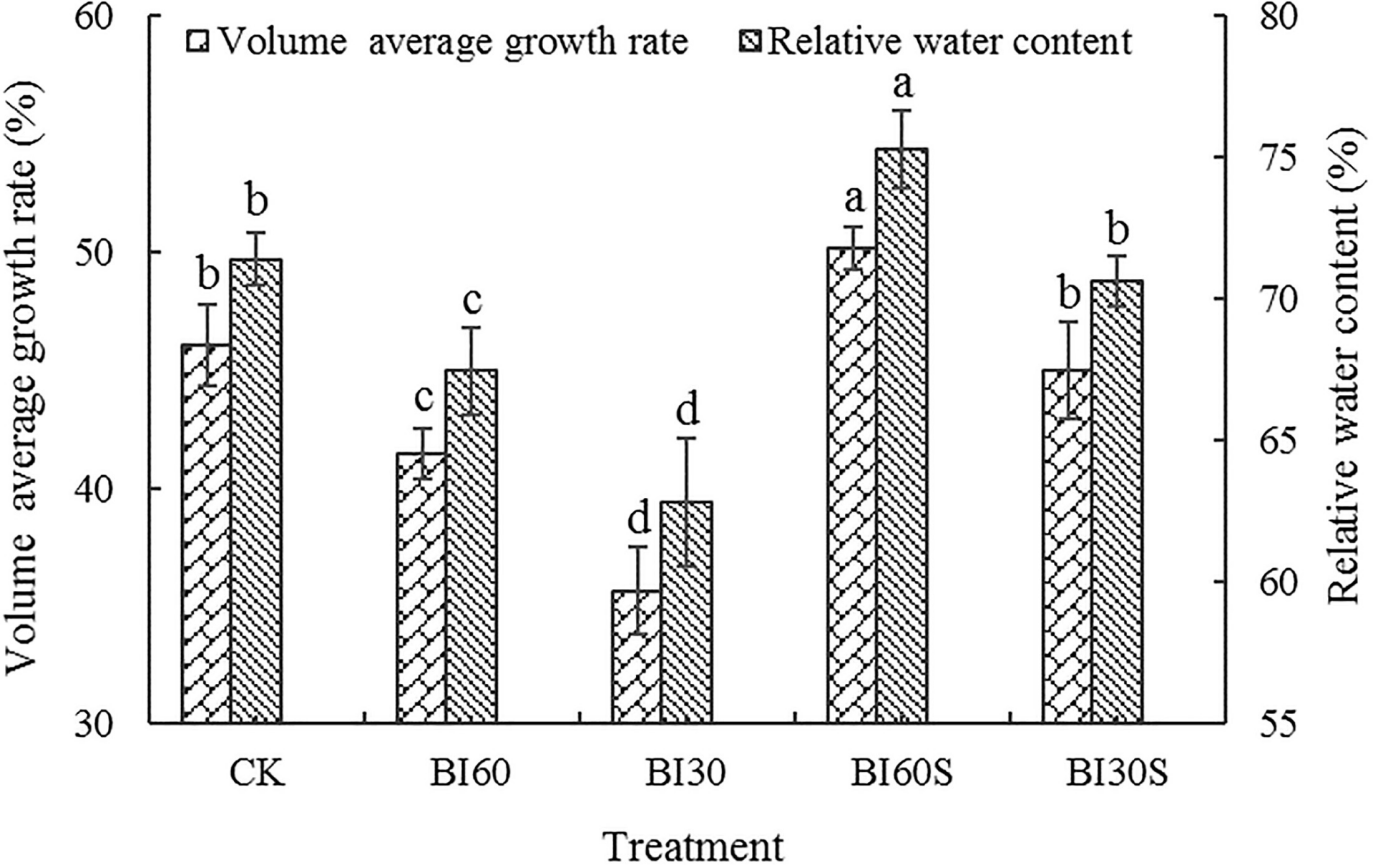
Effects of different irrigation treatments on the average growth rate in volume and relative water content in leaves of a poplar plantation. Bars are means, and error bars are standard deviations (n = 3). Means followed by same lowercase letter within each column were not significantly different among treatments (*P*>0.05).

The rank order of average volume growth rate in the five treatments was BI_60_+SAP > CK ≈ BI_30_+SAP > BI_60_ > BI_30_ (Fig 2). The average growth rate in the BI_60_+SAP treatment was the maximum, which was significantly increased compared with the other treatments. Although similar to CK, the average growth rate of the BI_30_+SAP treatment was evidently higher than that of either the BI_60_ or BI_30_ treatment, for which BI_60_ exhibited a higher growth rate than did BI_30_. Additionally, the relative water content in leaves of different treatments exhibited the same variation trend as the average volume growth rate; and the relative water content in the BI_60_+SAP treatment was higher than that in the other treatments. The data suggested that the combined use of reduced irrigation and super-absorbent polymers significantly facilitated the growth of poplar trees in the studied plantation.

## Discussion

The tree *Populus* × *euramericana* cv. ‘Neva’ is known as one of the most suitable species for wood production and afforestation in the arid and semi-arid districts of China [25, 34]. In this environment, water availability is the main factor suppressing plant growth and tree production. Since water is expected to become scarcer in the near future, water competition between plants and humans may make the situation even worse [12, 35]. Super-absorbent polymers have been successfully used as water-saving materials in horticulture and agriculture [4, 7, 13], but there are few reports of super-absorbent polymers use in poplar plantations. The incorporation of super-absorbent polymers into soil intensifies the retention of a great deal of water and nutrients that are released slowly, as required by the plant, thereby improving plant growth under conditions of limited water supply [3, 31], and also ameliorating the biological characteristics of the rhizosphere soil [7]. In the process of plant growth, the root system absorbs water and nutrients from the soil and also releases inorganic ions, secretes protons, and generates plentiful organic matter, all of which are added to the growth medium (called ‘root exudates’) and may have immediate or longer-term effects on organic matter accumulation and nutrient cycling in the soil-plant system [36]. In the present study, the 60% conventional border irrigation combined with super-absorbent polymers significantly increased the content of organic acids, amino acids, and total sugars in the root exudates. This result is likely because the applied super-absorbent polymers can lower soil bulk density, thereby increasing soil porosity and improving soil permeability [12, 37], which produces an ideal physical environment for tree root growth and thus facilitates the enhancement of root activity in poplar.

Many studies have used soil enzymes as indicators of soil microbial activity and fertility [38]. In our study, enzyme activities of invertase, urease, dehydrogenase and catalase under reduced irrigation were dramatically decreased. Similar results were also reported by Bastida et al. [6] who found that restricted irrigation had a negative impact on soil enzyme activities. In this experiment, the application of super-absorbent polymers likely compensated for the negative effects associated with restricted irrigation and were instead associated with an increase in soil enzyme activities under 60% irrigation. The positive effects of super-absorbent polymers on soil enzyme activity may be due to improvements in soil structure or increases in the amount of water retained near the roots which may have enhanced root activity and stimulated the release of more organic acids, amino acids, and total sugars. This increase in root exudates is supported by our results (Table 3) and may have facilitated increases in microbial biomass and activity, changes in the decomposition and mineralization rates of organic matter in the soil, and corresponding increases in enzyme activity [4]. Gianfreda [39] found that higher enzyme activity can be interpreted as a greater functional diversity of the microbial community in the rhizosphere soil.

In this study, the McIntosh index indicates the uniformity of the soil microbial community. The Shannon index provides information describing the distribution or spread of carbon source utilization by the microbial community [40]. Kennedy and Smith [41] also identified the Shannon index as a way of quantifying the evenness, richness, and diversity of the soil microbial community, whereas Staddon et al. [42] argued that the Shannon index is influenced more by species richness. In this study, the Shannon index for the 60% irrigation with super-absorbent polymers was obviously higher than that of other treatments. This result is probably because the application of super-absorbent polymers can alter the three phases of solid, liquid, and gas of soil, eventually forming a honeycomb-like structure, which increases the water-retaining property and nutrient-supplying capability of the soil to facilitate increased root activity; this, in turn, strengthens the connection between root metabolism and rhizosphere microorganisms [43]. Furthermore, such applications may significantly increase root exudates, which is the main source of carbon and energy for microorganisms in the rhizosphere soil. In addition, it can affect the solubility and effectiveness of the rhizosphere elements by changing the rhizosphere pH value [12], oxidation-reduction potential and chelation, which together influence microbial metabolism and microbial functional diversity in rhizosphere soil in a direct or indirect manner [22]. The Simpson index is weighted towards abundance of the most common species [42, 44, 45]. Our results demonstrated that the variation pattern of the Simpson index was opposite to the Shannon index or the McIntosh index, whereas Zhong and Cai [45] found that the pattern in variation of the Simpson index in a paddy soil was the same as that of the Shannon index for a long-term experiment. The differences between these studies concerning the relationship of the Simpson and Shannon indexes can be attributed to the different soil physicochemical properties, plant species, experimental period, and other factors [46, 47]. Therefore, among different irrigation measures, the 60% conventional border irrigation with super-absorbent polymers induced the highest increase in microbial diversity of poplar rhizosphere soil. Marinari et al. [48] observed that a greater functional diversity of microbial communities in the rhizosphere soil will result in elevated activities of many enzymes. Thus, this improvement of the microflora might also be related to the increase of enzyme activities found in the rhizosphere soil.

In the present study, the average growth rate in volume and relative water content in leaves following the 60% irrigation with super-absorbent polymers was significantly higher than that obtained in the other treatments. This result may be closely related to the improved root exudate content, increased microbial diversity, or elevated soil enzyme activities, all of which could augment soil fertility. Furthermore, a water-fertilizer combined micro-domain is introduced by super-absorbent polymers, which considerably improves the holding capacity of water and fertilizer to facilitate the synthesis of more biomass via photosynthesis [3, 12], further suggesting that the improvement of the micro-ecological environment in the vital root zone is beneficial to tree growth.

Moreover, we demonstrated that the effect of the 30% irrigation combined with super-absorbent polymers on soil enzyme activities, microbial diversity, and tree volume growth was significantly decreased compared with that of 60% irrigation coupled with super-absorbent polymers. The results further indicated that super-absorbent polymers cannot produce water and thus a sufficient water supply is still necessary for poplar management. This agreed with the findings of Bai et al. [12] and Han et al. [37], who reported that super-absorbent polymers require a minimum amount of water in order to provide benefits, suggesting that reaching a minimum threshold of water availability is a strong factor influencing both the microbial community and the efficacy of super-absorbent polymers. Our results also suggested that an appropriate reduction in the irrigation volume co-applied with super-absorbent polymers showed a noteworthy effect on the yield increase of poplar. These results highlighted the connections between enzyme activities and microbial diversity of soil and poplar productivity. Furthermore, the present study also underlined how a one-time application of super-absorbent polymers could guarantee a significant yield increase, at least in the following three years. We conclude that super-absorbent polymers are of great importance to both the water-saving strategy and high- yield cultivation of a poplar plantation, which also offers the advantage of saving on needless labor and time.

Additionally, in the actual use of super absorbent polymers, a variety of factors should be taken into account, such as the type of super absorbent polymers, application method, application amount, soil texture, water and fertilizer conditions and other factors [49]. At the same time, most of the super absorbent polymers are synthetic polymers, which are difficult to degrade or only partially degraded in the soil, and the polymers remaining in the soil are prone to cause soil environmental pollution [50].

## Conclusions

The results indicated that reduced border irrigation co-applied with super-absorbent polymers was effective in improving enzyme activities and microbial diversity in rhizosphere soil and enhancing poplar growth. Reduced irrigation with super-absorbent polymers, especially the 60% conventional border irrigation with super-absorbent polymers improved enzyme activities and microbial diversity of rhizosphere soil, and increased relative water content in leaves and volume growth rate. Furthermore, due to higher production, the 60% irrigation with super-absorbent polymers was better than the 30% irrigation with super-absorbent polymers. Therefore, the combined use of 60% conventional border irrigation with super-absorbent polymers ameliorated the micro-ecological environment of rhizosphere soil and improved poplar growth, showing real potential as a water-saving and high- yield cultivation measure in poplar plantations.
